# Protein disulfide isomerase A3 activity promotes extracellular accumulation of proteins relevant to basal breast cancer outcomes in human MDA-MB-A231 breast cancer cells

**DOI:** 10.1152/ajpcell.00445.2022

**Published:** 2022-11-14

**Authors:** Anna Germon, Kate J. Heesom, Reiss Amoah, Josephine C. Adams

**Affiliations:** ^1^School of Biochemistry, University of Bristol, Bristol, United Kingdom; ^2^University of Bristol Proteomics Facility, University of Bristol, Bristol, United Kingdom

**Keywords:** breast cancer, cell adhesion, extracellular matrix, proteomics, secretome

## Abstract

Metastasis and recurrence of breast cancer remain major causes of patient mortality, and there is an ongoing need to identify new therapeutic targets relevant to tumor invasion. Protein disulfide isomerase A3 (PDIA3) is a disulfide oxidoreductase and isomerase of the endoplasmic reticulum that has known extracellular substrates and has been correlated with aggressive breast cancers. We show that either prior PDIA3 inhibition by the disulfide isomerase inhibitor 16F16 or depletion of heparin-binding proteins strongly reduces the activity of conditioned medium (CM) of MDA-MB-231 human breast cancer cells to support promigratory cell spreading and F-actin organization by newly adherent MDA-MB-231 cells. Quantitative proteomics to investigate effects of 16F16 inhibition on heparin-binding proteins in the CM of MDA-MB-231 cells identified 80 proteins reproducibly decreased at least twofold (at *q* ≤ 0.05) after 16F16 treatment. By Gene Ontology analysis, many of these have roles in extracellular matrix (ECM) structure and function and cell adhesion; ribosomal proteins that also correlate with extracellular vesicles were also identified. Protein-protein interaction analysis showed that many of the extracellular proteins have known network interactions with each other. The predominant types of disulfide-bonded domains in the extracellular proteins contained β-hairpin folds, with the knottin fold the most common. From human breast cancer data sets, the extracellular proteins were found to correlate specifically with the basal subtype of breast cancer and their high expression in tumors correlated with reduced distant metastasis-free survival. These data provide new evidence that PDIA3 may be a relevant therapeutic target to alter properties of the ECM-associated microenvironment in basal breast cancer.

## Graphical abstract

**Figure:**
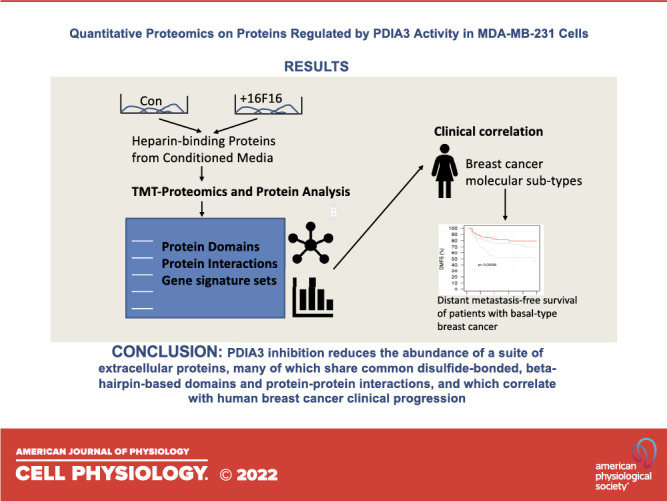

## INTRODUCTION

Despite improvements in population screening and early-stage treatments for some forms of human cancer, tumor recurrence and metastasis remain major causes of cancer mortality and there is an urgent need for new treatment options (1). It is well recognized that the tumor microenvironment (TME) makes major contributions to tumor progression by facilitating cancer cell adhesion, motility, invasion, and metastasis. Complex interactions between normal and tumor cells mutually drive properties of the TME and can result in recruitment of immune cells and altered phenotypic states of local tissue fibroblasts, alongside altered production of secreted factors and extracellular matrix (ECM), that collectively promote epithelial-mesenchymal transition (EMT) of tumor cells (reviewed in Refs. 2, 3). In addition, tissue microarchitecture can be altered through changes in the abundance, protein composition, or organizational state of ECM (4, 5). Changes to the ECM within the TME involve increased ECM stiffness, due to increased production of fibrillar collagens and/or their cross linking, along with elevated and chronic production of ECM components such as thrombospondins and tenascins that are normally transiently associated with tissue remodeling or wound repair (6–9). Because of the central role of ECM in cell interactions, tissue structure, and the pathogenesis of cancer, and because dense ECM also contributes to the chemo-resistance of malignant tumors (10, 11), approaches that could normalize ECM within the TME are of ongoing interest for potential new treatments against tumor invasion and metastasis. For example, chemical inhibitors of lysyl oxidase, the major extracellular enzyme that cross links fibrillar collagens, are under investigation (12).

Because ECM proteins typically undergo highly complex posttranslational modifications and processing before secretion, targeting of the intracellular steps of ECM production is also of interest as a route to modulate ECM composition or organization. In this regard, it is relevant to consider the protein disulfide isomerases (PDIs), a family of oxidoreductase enzymes that mostly reside within the endoplasmic reticulum (ER). These enzymes have roles in posttranslational folding/refolding of disulfide-bonded domains, such as characterize many ECM proteins (13, 14). Within the PDI family, PDIA3 (also known as ERp57) is of particular interest for its emerging association with progression of certain cancers. For example, proteomic analyses of breast cancers identified PDIA3 to be elevated in the tumors versus normal breast tissue (15, 16, 76) and to be upregulated in invasive ductal breast cancers compared with lobular cancers (17). PDIA3 has also been identified on the surface of tumor-associated macrophages in triple-negative breast cancer specimens (18) and has been suggested as a possible new prognostic marker (19).

In human breast cancer cell lines, knockdown of *PDIA3* transcripts reduced cell proliferation and increased cell sensitivity to treatment with chemotherapeutic agents or irradiation (20). PDIA3 was also found to be a driver of anchorage-independent growth of breast cancer cells in mammospheres (21). PDIA3 was required for the propensity of a metastatic subline of human MDA-MB-231 breast cancer cells for bone metastasis in a nude mouse model (22). Prior studies by this laboratory (23) have established that inhibition of protein disulfide isomerase activity in MDA-MB-231 cells by the PDI inhibitor 16F16, which preferentially inhibits PDIA3 (24), led to decreased cell spreading, reduced numbers of focal adhesions, and inhibited cell migration in vitro. We have also established by comparative studies of mouse embryo fibroblasts (MEFs) that gene knockout of *Pdia3* correlated with reduced cell spreading relative to wild-type MEFs (WT-MEFs) and strongly affects the secretome of MEFs, resulting in reduced extracellular abundance of a number of heparin-binding proteins in the serum-free conditioned medium (CM) of *Pdia3*^−/−^ MEFs compared with the CM of WT-MEFs. The affected proteins included several ECM proteins and growth factors (25). In addition, whereas exposure to CM of WT-MEFs restored cell spreading of *Pdia3*^−/−^ MEFs, the CM of *Pdia3*^−/−^ MEFs poorly supported adhesion, F-actin organization, and focal adhesion formation by WT-MEFs (25).

With regard to the effects of PDIA3 inhibition on promigratory phenotypes of breast cancer cells (23) and in consideration of PDIA3 as a prospective therapeutic target in breast cancer, we report here a comparative proteomic analysis to identify effects of 16F16 inhibition on the secreted, heparin-binding proteins produced by MDA-MB-231 cells. Bioinformatic analyses of the identified proteins, their protein-protein interaction networks, and their domains demonstrate enrichment for extracellular proteins containing disulfide-bonded β-hairpin domains and functional enrichment for proteins with roles in ECM, cell adhesion, and epithelial-mesenchymal transition (EMT). Analysis against human breast cancer tumor data sets implicates a clinical relevance of PDIA3 and the identified extracellular proteins to the basal subtype of breast cancer and to distant metastasis-free survival (DMFS) outcomes. These results provide new evidence for PDIA3 as a prospective relevant therapeutic target in breast cancer.

## METHODS

### Materials

Chemicals and other materials were obtained from Sigma unless otherwise stated. Details and catalog numbers of reagents and plasticware are given in Table 1. MDA-MB-231 human female breast cancer cells (described originally in Ref. 26) were purchased from American Type Culture Collection and cultured in high-glucose Dulbecco’s modified Eagle’s medium (DMEM) (which contains 44.5 µM phenol red), supplemented with 10% fetal bovine serum (FBS) at 37°C in a humidified, temperature-controlled Heracell 150 incubator (Thermo Scientific) gassed with 5% CO_2_. For some experiments, cells were washed through two changes of serum-free Fibroblast Growth Medium (FGM; Promega), which contains 3.3 μM phenol red, and the experiments were carried out in this medium. Cell growth properties were indistinguishable between the media. Cells were passaged by brief washing and incubation in trypsin-EDTA followed by washing in serum-free medium, counting, and replating as needed. Antibodies used are listed in Table 2.

**Table 1.** Details of reagents and plasticware used

Item	Supplier	Catalog No.
100-mm TC-treated cell culture dishes	Falcon	353003
15-mL centrifuge tube	Sarstedt	62.554.002
16F16 inhibitor	Sigma	SML-0021
50-mL polypropylene conical tube	Falcon	352070
Six-well Cell Clear Flat Bottom TC-treated Multiwell Culture Plate	Falcon	353934
60-mm TC-treated cell culture dishes	Falcon	353002
Acrylamide (40%)	Bio-Rad	1610140
Ammonium persulfate powder (APS)	BDH (now VWR Life Science)	BDH9214-500G
20- to 200-μL sterile filter tips	Anachem	ABA2005
Bis-acrylamide (2%)	Bio-Rad	161-0142
Bromophenol blue	Sigma	B0126
Cell scraper	Falcon	35386
Dimethyl sulfoxide (DMSO)	GPR	282164K
dl-dithiothreitol (DTT)	Sigma	D9779
Dulbecco’s modified Eagle’s medium	Sigma-Aldrich	D6429
Enhanced chemiluminescence (ECL) WB detection agent	Amersham	RPN2209
Ethanol (100%)	Sigma-Aldrich	32221
Ethylenediaminetetracetic acid (EDTA)	Sigma	ED2SS
Fibroblast Growth Medium	Promocell	C-23010
Fetal bovine serum	Sigma-Aldrich	F7524
Glycerol	Sigma	G5516
Glycine	Sigma	G8898
Heparin-agarose in aqueous ethanol suspension	Sigma-Aldrich	H0402
Hydrochloric acid	Sigma	H1758
l-ascorbic acid	Sigma	A4544
Methanol	Sigma	322415
Milk powder	Sainsbury’s	7742528
*N*,*N*,*N*′, *N*′-tetramethylethylenediamine (TEMED)	Sigma	T9281
Nunc Cell Culture Treated Flasks with Filter Caps	Thermo Scientific	136196
Paraformaldehyde (PFA) 16% (wt/vol)	Alfa Aesar	43368
Phalloidin-Atto 565	Sigma	94072
Pierce Control Agarose Resin	Thermo Scientific	26150
Pierce Protease Inhibitor Tablets-Mini	ThermoFisher Scientific	88665
Ponceau S stain	Sigma	P71701
Precision Plus Protein Dual Color Standards	Bio-Rad	1610374
PVDF (0.2 μm) membrane	Amersham	10600021
Slab gel casting stand	ATTO Corporation	AE6200
Sodium dodecyl sulfate (SDS)	Fischer Scientific	28906
Syringe filter unit, 0.22 μm, polyethersulfone, 33 mm, gamma sterilized	Millex	SLGP033RB
Tissue culture treated flasks 250 mL	Falcon	35316
Transfer pipettes	Alpha Laboratories	LW4141
Triton X-100	Sigma	T6066
Trizma base (Tris)	Sigma	T6066
Trypsin-EDTA	Sigma-Aldrich	T3924
Tween 20	Sigma	P2287
VectaMount	Vector	H5000
VectaShield with 4’,6’-diamidino-2-phenylindole	Vector	H1200

**Table 2.** Details of primary and secondary antibodies used

				Dilution		
Antigen	Antibody	Supplier	Catalog No.	WB	IF	RRID
Binding immunoglobulin protein (BiP)	Rabbit polyclonal IgG	Abcam	Ab21685	1:2,000		AB_2119834
Fibronectin (FN)	Rabbit polyclonal IgG	Sigma	F3648	1:600		AB_476976
Glyceraldehyde-3-phosphate dehydrogenase (GAPDH)	Mouse monoclonal IgG	Abcam	Ab9484	1:600		AB_307274
Insulin growth factor binding protein-7(IGFBP7)	Rabbit polyclonal IgG	Abcam	Ab74169	1:1,000		AB_1860675
Lysyl oxidase-like 2 (LOXL2)	Rabbit polyclonal IgG	Abcam	Ab96233			AB_10677617
Protein disulfide isomerase (PDIA3/Erp57)	Mouse monoclonal IgG1	Abcam	Ab13506	1:2,000		AB_1140700
Vinculin	Mouse monoclonal IgG	Sigma	V4505		1:300	AB_477617
Mouse IgG	Alexa Fluor 488-conjugated polyclonal goat IgG	Life Technologies	A11001		1:200	AB_2534069
Mouse IgG, IgA, IgM	HRP-conjugated goat IgG	LI-COR	926-80010	1:50,000		AB_2721263
Rabbit IgG, IgA, IgM	HRP-conjugated goat IgG	LI-COR	926-80011	1:50,000		AB_2721264

HRP, horseradish peroxidase; IF, immunofluorescence; WB, Western blot.

### Preparation of Cell Lysates and Conditioned Media

MDA-MB-231 cells trypsinized from stock culture were pelleted by centrifugation and washed twice in 8 mL of FGM. For experiments, p90 tissue culture dishes were set up, each containing 1.5 × 10^6^ cells in a total volume of 8 mL of FGM. l-ascorbic acid was added from a 10 mg/mL stock to give a final concentration of 50 µg/mL to promote collagen synthesis (27). Cultures were incubated at 37°C for 2–3 h to allow cells to attach, and then 16F16 inhibitor was added at 5 μM final concentration, previously established to be effective and nontoxic (23). Control dishes received an equivalent volume of DMSO as solvent-only control [0.1% (vol/vol) final dilution, corresponding to a final DMSO concentration of 8.46 mM]. All dishes were incubated for 48 h at 37°C and then processed according to the exact experimental design. For preparation of cell extracts, conditioned medium (CM) was removed, the cell layer gently rinsed three times in phosphate-buffered saline (PBS), and 200 μL of hot sodium dodecyl sulfate-polyacrylamide gel electrophoresis (SDS-PAGE) sample buffer containing 100 mM dithiothreitol (DTT) (referred to subsequently as SB+DTT) added per dish. The cell lysate was scraped into a sterile 1.5-mL Eppendorf tube and stored at −20°C. For preparation of CM after 48 h, medium from each p90 dish was put in a 50-mL polypropylene conical tube (Fisher) and centrifuged at 1,000 rpm for 5 min to pellet cell debris. Supernatants were recovered and filtered through a 0.22-μm-pore syringe filter unit (Millipore) into a fresh 50-mL polypropylene conical tube for use in experiments. For depletion of heparin-binding proteins, the filtered CM was incubated with 15-µL (vol/vol) heparin-agarose beads or 15-µL agarose-only control beads for 1 h with rotation at 4°C. Beads were then collected by centrifugation, and the supernatants were used in experiments.

### Fluorescence Microscopy: Effect of the PDIA3-Dependent Secretome on Cell Spreading

Stock MDA-MB-231 cells were trypsinized, washed twice in FGM, and plated at 1.5 × 10^5^ cells on sterile glass coverslips in wells of six-well cell culture trays (Costar) containing either 3 mL of FGM or 3 mL of CM (prepared as described above). Trays were incubated at 37°C for either 4 h or 20 h to allow cells to attach and spread. At each time point, medium was gently removed from each well, the wells were gently washed three times in PBS to remove nonadherent cells, and the adherent cells were then fixed in 4% paraformaldehyde (PFA) in PBS at room temperature (RT) for 10 min. Wells were washed three times in PBS and stored for a maximum of 3 days at 4°C before cell permeabilization and staining.

### Fluorescence Microscopy: Quantification of Cell Areas

Fixed cells on glass coverslips were permeabilized with 0.5% Triton X-100 in PBS for 10 min and then reimmersed in PBS. Coverslips were drained and placed cell side up in a humidified chamber, and cells were stained with phalloidin-Atto 565 (Table 1) diluted 1:100 in PBS for 50 min at RT to visualize F-actin. Coverslips were rinsed three times in a bath of PBS, and then in deionized water to remove salts, and mounted cell side down in VectaShield containing 4′,6-diamidino-2-phenylindole (DAPI) (Vector Labs) on glass microscope slides. All samples were examined under a Leica SP5-AOBS confocal laser scanning microscope attached to a Leica DM I6000 inverted epifluorescence microscope with an HCX PL APO lambda blue ×63 1.4 NA oil objective. With Leica Application Suite AF software 2.7.3.9723, *z*-stack images were taken at ∼0.50-μm *z*-slice thickness from the base of the cells upward, over a 2.5- to 3-μm total *z*-stack thickness. A 20-mW solid-state yellow laser (561 nm) was used to detect phalloidin-Atto 565 and a 50-mW 405-nm diode laser for detection of DAPI. Three coverslips were imaged per condition per experiment, and *z*-stack images were collected such that at least 50 cells could be scored. All files were saved as TIFFs and loaded into ImageJ (RRID:SCR_003070) as merged DAPI and phalloidin images of each section. Each *z* stack of sections was merged with Image> Stacks> Z-Project (Max Projection). Stacked images were processed with Image> Adjust> Threshold following Process> Binary> Make Binary. The areas of cells were measured with Analyze> Analyze Particles. These steps were combined into an automated macro in which cell areas were extracted and added to the Region of Interest (ROI) manager. Results were also inspected manually, and some cell areas were remeasured with the ImageJ freehand outline tool for random checks or if the macro failed to outline a cell correctly. Three independent experiments were conducted unless stated otherwise, and every experiment included the control and test conditions.

### Large-Scale Heparin-Affinity Pulldown of Conditioned Media

For large-scale collection of CM for tandem mass tagging (TMT) proteomics, eight p90 dishes were set up as described above. Four dishes were treated with 16F16 and four with DMSO only. After 48 h, the CMs for each experimental condition were pooled (giving a total of 32 mL of CM per condition), centrifuged to remove cell debris, and sterile filtered through a 150-mL Nalgene Stericup filtration unit with Durapore [polyvinylidene difluoride (PVDF)] low-protein-binding membrane. Each filtered CM was added to a fresh 50-mL polypropylene conical tube, and three tablets of Pierce Protease Inhibitor Tablets-Mini (Table 1) were added. A total of 160 µL of heparin-agarose beads and 230 µL of control agarose beads were prepared according to manufacturer’s instructions. Each CM sample was incubated with 100 µL [of 1:1 (vol/vol) suspension] of control agarose beads, to absorb any nonspecific agarose-binding proteins, on a rotatory wheel for 1 h at 4°C. Tubes were then centrifuged at 1,000 rpm for 5 min to pellet the beads. Each supernatant CM was then transferred to a fresh 50-mL tube, and 80 µL of heparin beads in Tris-buffered saline (TBS) [1:1 (vol/vol) suspension] was added to each tube. Tubes were placed on a rotating wheel at 4°C for 1.5 h, to allow heparin-binding proteins to attach to the heparin-agarose beads. Each suspension was then centrifuged at 1,000 rpm for 5 min to pellet the beads. Beads were washed three times in TBS and then moved to 1.5-mL Eppendorf tubes and rinsed once more, and all liquid was removed from the final pellets. Fifty microliters of SB+DTT was added to each tube and boiled to dissociate the bound proteins. Samples were then centrifuged at 10,000 rpm for 30 s and stored at −20°C before proteomics analysis. Four independent experiments were carried out.

### Tandem Mass Tag-Based Quantitative Proteomics

For proteomics, the samples from each experiment were resolved in one dimension on a SDS-PAGE gel to facilitate removal of buffer components that would interfere with downstream liquid chromatography-mass spectrometry (LCMS) analysis. Electrophoresis was carried out until the dye front had moved ∼1 cm into the resolving gel. Each gel lane was then excised, and the proteins in each gel slice were digested with trypsin (which cleaves COOH terminal to lysine and arginine residues) with a DigestPro automated digestion unit. Resulting peptides were extracted and labeled with Thermo Fisher Scientific TMT reagent (catalog no. 90113) according to the manufacturer’s protocol. Free tags were quenched with hydroxylamine, and then all labeled peptide samples were pooled. The pooled material was analyzed by LCMS using an Orbitrap Fusion Tribrid mass spectrometer with data analysis performed with Thermo Scientific Proteome Discoverer software. Data from the four experiments underwent statistical analysis by Dr. Phil Lewis, University of Bristol Proteomics Facility. Statistical analysis was performed only on the proteins identified from all four independent experiments. Each protein abundance was brought closer to a normal distribution by log2 transformation. Because of the biological and technical variance between replicates, a paired *t* test was used to identify meaningful (*P* ≤ 0.05) biological changes between the control and +16F16 conditions. Volcano plots were prepared in R.

### SDS-PAGE and Immunoblotting

Gels were cast as 10% polyacrylamide SDS-PAGE resolving gels overlain with 4% polyacrylamide stacking gel. Before gel loading, samples were heated at 95°C for 5 min. Samples that contained heparin-agarose or control agarose beads were centrifuged at 1,000 rpm for 1 min to pellet beads and the supernatants loaded. Precision Plus Protein Dual Color Standards (Table 1) provided molecular weight reference markers on each gel. Proteins were stacked at 80 V for 1 h and then separated in the resolving gel at 140 V for 4 h. For immunoblotting, proteins were transferred onto 0.2-µm-pore polyvinylidene difluoride (PVDF) membrane (Millipore) at 15 V for 1.5 h in a Trans-Blot SD Semi-Dry Transfer Cell (Bio-Rad). The membrane was stained with Ponceau S to visualize transferred proteins and washed three times in deionized H_2_O to increase band clarity, and a digital image was taken in a G:BOX Chemi XRQ with Genesys software (both from Syngene). The membrane was then cut into segments according to the experimental design and blocked for 30 min in immunoblot blocking buffer (BB) composed of PBS containing 2% (wt/vol) semiskimmed dried milk and 0.2% (vol/vol) Tween 20. Specific primary antibodies (Table 2) were diluted in BB and incubated with membranes at 4°C overnight with rotation. Membranes were then washed three times for 10 min each in BB and incubated with the appropriate diluted secondary antibody (Table 2) for 1 h at RT with rotation. After washing three times for 10 min in BB and two 10-min washes in PBS, bound antibodies were visualized with Amersham enhanced chemiluminescence (ECL) Western blot detection reagent (Table 1) and imaged in a G:BOX Chemi XRQ. The TIFF digital image file was exported to GeneTools (Syngene) for quantitative band analysis and normalization to loading control.

### Bioinformatics Analyses of PDIA3-Dependent, Heparin-Binding Proteins

All proteins identified by TMT proteomics to undergo an at least twofold change in abundance and found to be significantly (*P* ≤ 0.05) different between the control and 16F16 samples across the four independent experiments were filtered to satisfy a false discovery rate (FDR) of 5%. Proteins with FDR of <0.05 (*q* ≤ 0.05) were taken forward for further investigation. Accession numbers were converted to gene names with https://www.uniprot.org/uploadlists/. If the gene name was not found automatically, it was found manually in the UniProt Knowledge Base/SwissProt (RRID:SCR_021164, https://www.uniprot.org/) (28). Proteins were analyzed for known intracellular or extracellular location, initially according to UniProt and then by analysis for the presence of a secretory signal peptide or transmembrane domain within the protein sequence using SIGNALP 6.0 (29) and TMHMM 2.0 (30) through the online bioinformatics resources of the Technical University of Denmark (https://services.healthtech.dtu.dk).

#### Gene Ontology analysis.

The complete list of 80 proteins and the subsets of extracellular or intracellular proteins were each analyzed for enrichment in Gene Ontology (GO) terms (31, 32) according to the GO categories of Biological Process, Molecular Function, and Cellular Component. Searches were carried out through the resources of the Protein Annotation Through Evolutionary Relationship (PANTHER) Classification System, version 17.0, (RRID:SCR_004869; http://pantherdb.org) (33), using the analysis tool “statistical over-representation test” against *Homo sapiens* reference genes at default parameters. A FDR *q* value of <0.05 was considered significant. Outputs were ranked by FDR *q* value, and the top 10 significant results for each category were tabulated. A separate analysis for enriched GO terms was made with the Gene Ontology Enrichment Analysis and Visualization tool (GOrilla) (RRID:SCR_006848; http://cbl-gorilla.cs.technion.ac.il/) (34). In this analysis, the background protein list used was the full list of proteins detected by the TMT-based quantitative proteomics (345 proteins). Outputs from GOrilla were saved as jpg files.

#### Domain identification and analysis.

NP_prefixed reference protein sequences were retrieved from the National Center for Biotechnology Information (NCBI) (RRID:SCR_006472, https://www.ncbi.nlm.nih.gov/protein/) GenPept database, and the sequence features of each polypeptide as stated under “Ref Seq attributes” were examined. Each polypeptide sequence in FASTA format was analyzed for domain composition in InterProScan (RRID:SCR_005829, https://www.ebi.ac.uk/interpro/search/sequence/) (35) at default settings. Identified disulfide bonds or protein domains that contain disulfide bonds, along with their superfamily associations from InterProScan, were listed for each protein in a spreadsheet. Protein domain composition was also analyzed independently against the NCBI Conserved Domain Database (CDD, RRID:SCR_002077, https://www.ncbi.nlm.nih.gov/Structure/cdd/cddsrv) (36), and results were recorded in the Excel spreadsheet. To identify the structural fold families to which the disulfide-bonded domains belonged, the protein superfamilies identified by InterProScan and CDD were searched in the Structural Classification of Proteins database (SCOP2, RRID:SCR_007039, https://scop.mrc-lmb.cam.ac.uk/) (37) and in UniProtKB, and parent folds containing conserved disulfide bonds were identified. As needed, additional information was obtained on fold structures from sources cited in the databases. From these classifications, proteins from the proteomics data set were grouped into their respective fold superfamilies. Schematic diagrams of fold types were prepared in Word based on data from CDD and known structures cited in the databases.

#### Protein networks analysis.

Potential interactions between proteins within the data set were examined by STRING Protein-Protein Interaction Networks Functional Enrichment Analysis (RRID:SCR_005223, https://string-db.org/) (38), using the parameters interaction sources, text mining, experiments, and databases with a high confidence threshold of 0.700 and display of only the query proteins in the output network. Analyses were made for the complete data set of 80 proteins and, separately, for the extracellular or intracellular proteins from the data set. Outputs from STRING were saved as JPEG files for visual interpretation.

#### Gene signature analysis.

Searches of Gene Set Enrichment Analysis (GSEA) (RRID:SCR_003199, https://www.gsea-msigdb.org/gsea/index.jsp) (39, 40) were carried out at the Molecular Signatures Database (MSigDB; RRID:SCR_016863) to investigate representation (by gene name) in previously identified gene signature sets in the GSEA “Hallmark” category. A FDR *q* value of <0.05 was taken as significant, and the top 10 FDR *q* value results were tabulated.

### Correlation with Human Breast Cancer

Expression of *PDIA3* in human breast cancers was assessed in TNMplot (https://tnmplot.com/analysis/) (41) by using the tool “gene expression comparison” to compare *PDIA3* expression, as measured by RNASeq, in invasive breast cancers (*n* = 1,097) versus normal tissue (*n* = 405). The tool includes statistical analysis by Mann–Whitney *U* test. Correlation of *PDIA3* expression with expression of individual transcripts encoding the identified extracellular proteins was examined with the TNMplot tool “correlation analysis” as applied to RNASeq data from invasive breast cancer tumors; only correlations with *P* value < 0.05 are reported in the figure. Expression of PDIA3 or the set of identified extracellular proteins in breast cancer tumor subtypes and the possible relationship of the set to distant metastasis-free survival over 10 yr were examined through the resources of the “Gene expression-based Outcome for Breast Cancer Online” (GOBO) database (http://co.bmc.lu.se/gobo/) (42). This analysis was conducted against all tumor samples (*n* = 1,881) in the database, using gene names as in GOBO. For transcripts encoding the extracellular proteins, a set of 45 was examined, as *CLSTN1*, *COL12A1*, *MST1*, *NOG*, and *SOD3* were not included in the GOBO database. A separate analysis was conducted for *PDIA3* transcripts alone. Visual outputs from GOBO v1.0.3 were saved as pdf files.

### Statistical Analysis

For the laboratory experiments, tests of statistical significance were performed in GraphPad Prism 9.0 (RRID:SCR_002798) except for the analysis of proteomics data (see *Tandem Mass Tag-Based Quantitative Proteomics*). Shapiro–Wilks normality tests for the cell area data sets and calculation of descriptive statistics for all data sets were carried out in Prism. Experiments involving two experimental conditions and equal variance were analyzed by unpaired Student’s *t* test; Welch’s correction was applied if variances were significantly different. Nonparametric data were analyzed by Mann–Whitney *U* test. Experiments involving more than two experimental conditions and nonparametric data were analyzed in Prism by Kruskal–Wallis test with Dunn’s multiple comparison test; parametric data were analyzed by two-way ANOVA with Tukey’s multiple comparison post hoc testing.

## RESULTS

### Promotion of Cell Spreading by MDA-MB-231 Conditioned Medium Depends on PDIA3 Activity or Heparin-Binding Proteins

**Figure 1.**
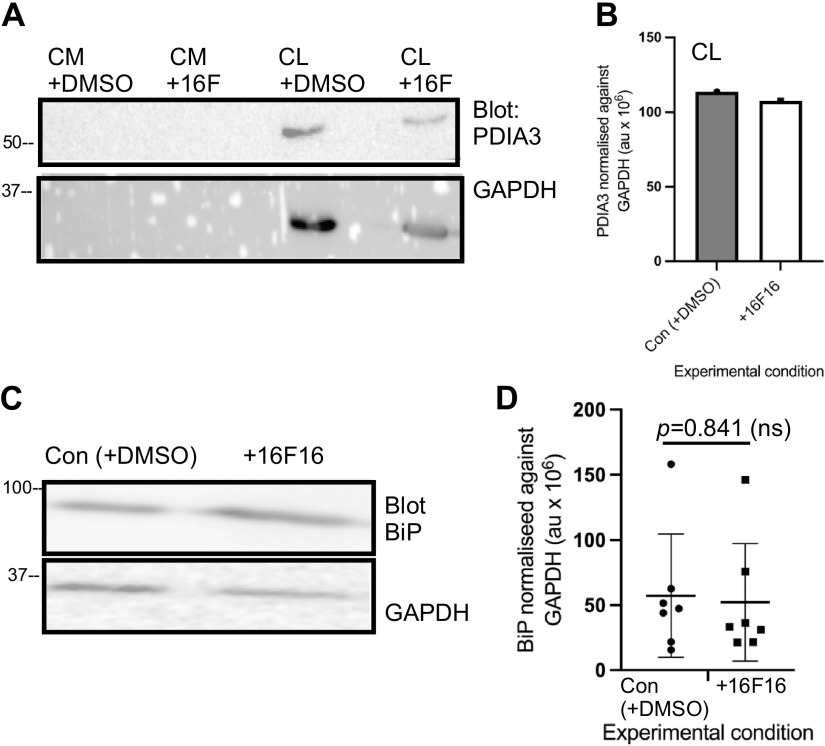
Effects of 16F16 inhibition on abundance of PDIA3 and BiP in MDA-MB-231 cells. *A* and *C*: immunoblots of PDIA3 (*A*) and BiP (*C*) from conditioned medium (CM) or whole cell lysates (CL) of MDA-MB-231 cells treated with 16F16 inhibitor (+16 F) or DMSO solvent control (+DMSO) for 48 h. Proteins were resolved on 10% polyacrylamide gels under reducing conditions, transferred to PVDF for immunoblotting with the indicated antibodies, and then stripped and reprobed for GAPDH as a loading control. Molecular mass markers are in kDa. *B* and *D*: quantified analysis of PDIA3 or BiP in CL, normalized against GAPDH. Statistical analysis in *D* by unpaired *t* test. Each data point represents 1 experiment; horizontal bars show the mean and SD. au, Arbitrary units; ns, not significant.

We utilized MDA-MB-231 cells as a well-established in vitro model of invasive human breast cancer cells with similarity to the basal subtype of breast cancer that has undergone epithelial-to-mesenchymal transition (43). The inhibitor 16F16 acts on PDIA3 and, to a lesser extent, on PDIA1 (24). We first examined whether inhibition with 16F16 over a 48-h period (the time period used to produce CM in subsequent experiments) altered the abundance of PDIA3 protein. As demonstrated by Western blot of whole cell lysates, PDIA3 protein was only marginally decreased (Fig. 1, *A* and *B*). In view of conflicting reports on a role of PDIA3 in inhibiting ER stress (44, 45), we also compared the abundance of GRP78/BiP, an ER-resident chaperone protein that has functions in the unfolded protein response (46). BiP was clearly present in both the control and 16F16-treated cells, and its abundance was not increased in the presence of 16F16 (Fig. 1, *C* and *D*).

**Figure 2.**
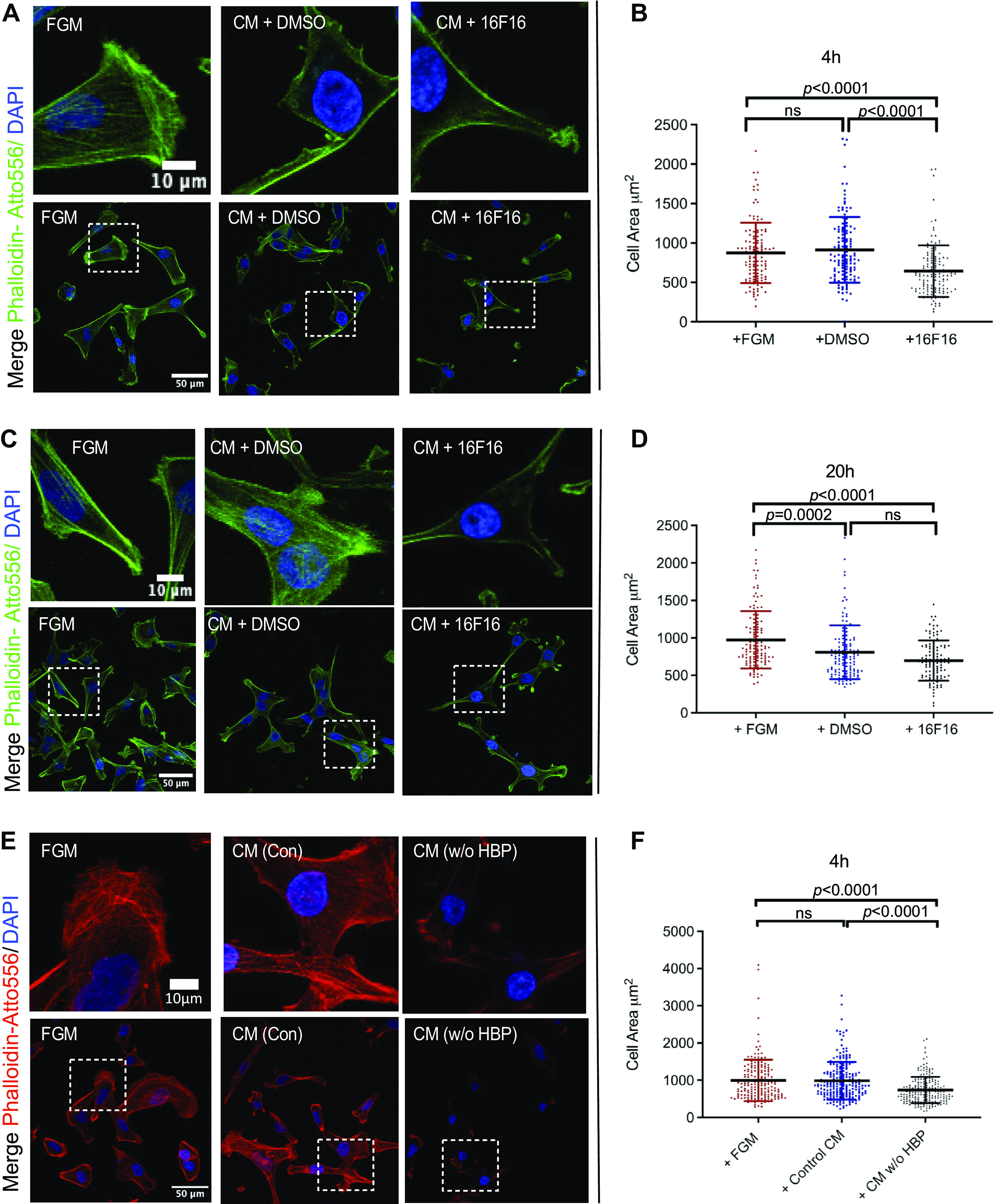
Effects of 16F16 inhibition or depletion of heparin-binding proteins on stimulation of spreading and F-actin organization by conditioned medium (CM) from MDA-MB-231 cells. *A–D*: comparison of effects of fresh Fibroblast Growth Medium (FGM) or CM from control (+DMSO) or treated (+16F16) cells on F-actin organization (*A* and *C*) and cell spreading (*B* and *D*) in newly plated MDA-MB-231 cells after adhesion for 4 h (*A* and *B*) or 20 h (*C* and *D*). *E* and *F*: effects of depletion of heparin-binding proteins (HBP) from CM on its activity to support F-actin organization (*E*) and cell spreading (*F*) of newly plated MDA-MB-231 cells. The adhesion time was 4 h. In *A*, *C*, and *E*, the box areas at *bottom* are shown enlarged at *top*. *B*, *D*, and *F*: statistical analysis by Kruskal–Wallis test with Dunn’s multiple comparison test. ns, Not significant.

We next compared functional activities of CM produced by control MDA-MB-231 cells with CM from cells treated with 16F16 inhibitor. Prior experiments have indicated that the serum-free CM from *Pdia3*^−/−^ MEFs does not support cell spreading and F-actin organization by MDA-MB-231 cells to the same extent as serum-free CM from wild-type MEFs (WT-MEFs) (23). This suggested that proteins that depend on PDIA3 in the secretory pathway for their posttranslational processing and secretion may function to support cell spreading after secretion. However, a limitation of the prior study was that the experimental materials were cross-species (i.e., effects of mouse cell CM tested on human cells). We prepared serum-free CM from control or 16F16-treated MDA-MB-231 cells, and each CM was tested for its activity to promote the spreading of naive, newly plated MDA-MB-231 cells. After 4 h of cell attachment, cells incubated in control CM had more prominent F-actin bundles in the cell cortex and cell body compared with the cells exposed to 16F16-treated CM (Fig. 2*A*). This morphological difference was also apparent quantitatively: the spread areas of the cells exposed to CM from 16F16-treated cells were reduced compared with the areas of control cells (Fig. 2*B*). Differences in F-actin organization were maintained up to at least 20 h of cell attachment (Fig. 2*C*), and at 20 h cells exposed to CM from 16F16-treated cells had smaller areas than cells exposed to control CM (Fig. 2*D*). However, at the 20 h time point cells exposed to CM containing DMSO also showed reduced areas compared with cells plated in fresh FGM, perhaps due to effects of cell metabolites within the CM (Fig. 2*D*).

To further investigate the functional properties of CM, we investigated the role of heparin-binding proteins in the cell-spreading activity of CM from control MDA-MB-231 cells. Many secreted proteins that have roles in cell attachment and cell spreading contain heparin-binding domains (47), and we have shown that heparin-binding proteins are essential for the stimulation of spreading of *Pdia3*^−/−^ MEFs by CM from WT-MEFs (25). Serum-free CM was produced from control MDA-MB-231 cells as above and then mixed with either control agarose beads or heparin-agarose beads, the beads removed by centrifugation and filtration, and the residual supernatants tested in MDA-MB-231 cell spreading for 4 h. The CM depleted for heparin-binding proteins was markedly less effective at supporting MDA-MB-231 cell spreading assays and F-actin organization than the control CM or fresh FGM, as apparent from cell morphology (Fig. 2*E*) and cell areas (Fig. 2*F*). Our quantitative proteomics results (see below) demonstrate the efficacy of the heparin-agarose treatment in depleting known heparin-binding proteins such as fibronectin (FN) and thrombospondin1 (TSP1).

### Identification of Heparin-Binding, PD1A3-Dependent Proteins in Conditioned Medium of MDA-MB-A231 Cells

Because either 16F16 inhibition or depletion of heparin-binding proteins reduced the activity of MDA-MB-231 CM to support cell spreading, we undertook quantitative proteomics to identify which heparin-binding proteins depend on protein disulfide isomerase activity for their abundance in CM. Identification of proteins was carried out by tandem mass tagging (TMT) quantitative proteomics on large-scale preparations of the heparin-binding fractions of serum-free CM from either DMSO-treated (control) or 16F16-treated MDA-MB-231 cells. Four independent experiments were carried out. From the lists of proteins positively identified in each experiment, a list of the proteins consistently identified by all four independent experiments was made (*n* = 345 proteins; Supplemental Table S1). The abundance of each of these proteins in the +DMSO versus +16F16 conditions was compared by paired Student’s *t* test, and the significance was normalized (−log10*P* value) around zero. Proteins either increased or decreased in abundance by at least twofold were then itemized for further analysis.

**Figure 3.**
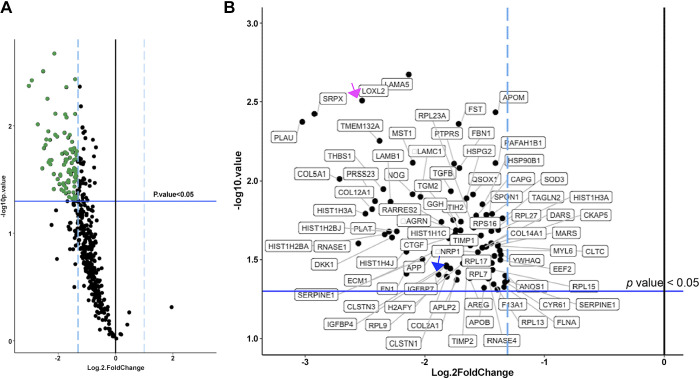
Results of tandem mass tagging (TMT)-quantitative proteomics to identify heparin-binding proteins of altered abundance in the conditioned medium (CM) of MDA-MB-231 cells after 16F16 treatment. *A*: volcano plot of proteins reproducibly altered in abundance by 16F16 treatment of cells. Based on TMT proteomics of CM from control and 16F16-treated cells and 4 independent experiments. Vertical dashed lines indicate ≥2-fold changes in protein abundance; blue horizontal line indicates *P* = 0.05 threshold for significance. Each dot represents a single protein; green dots show the proteins decreased ≥2-fold at *P* ≤ 0.05 by 16F16 treatment. *B*: enlarged view of the significantly decreased (*q* value <0.05) proteins, with each protein identified by gene name. Magenta and blue arrows indicate the 2 proteins chosen for validation by immunoblotting. See Supplemental Tables S1 and S2 for details of the proteins.

Whereas no proteins were significantly increased by 16F16 treatment according to these criteria, 107 proteins were at least twofold decreased in abundance with a *P* value ≤ 0.05 (Fig. 3*A*; green data points on volcano plot) The list of 107 proteins was then filtered to remove potential false positives such that the proteins with a false discovery rate (FDR) of *q* ≤ 0.05 were retained. This resulted in a set of 80 proteins that constituted the PDIA3-dependent, heparin-binding secretome and that we refer to here as the proteomics data set (Fig. 3*B*; proteins are plotted according to *P* values as in Fig. 3*A*).

**Figure 4.**
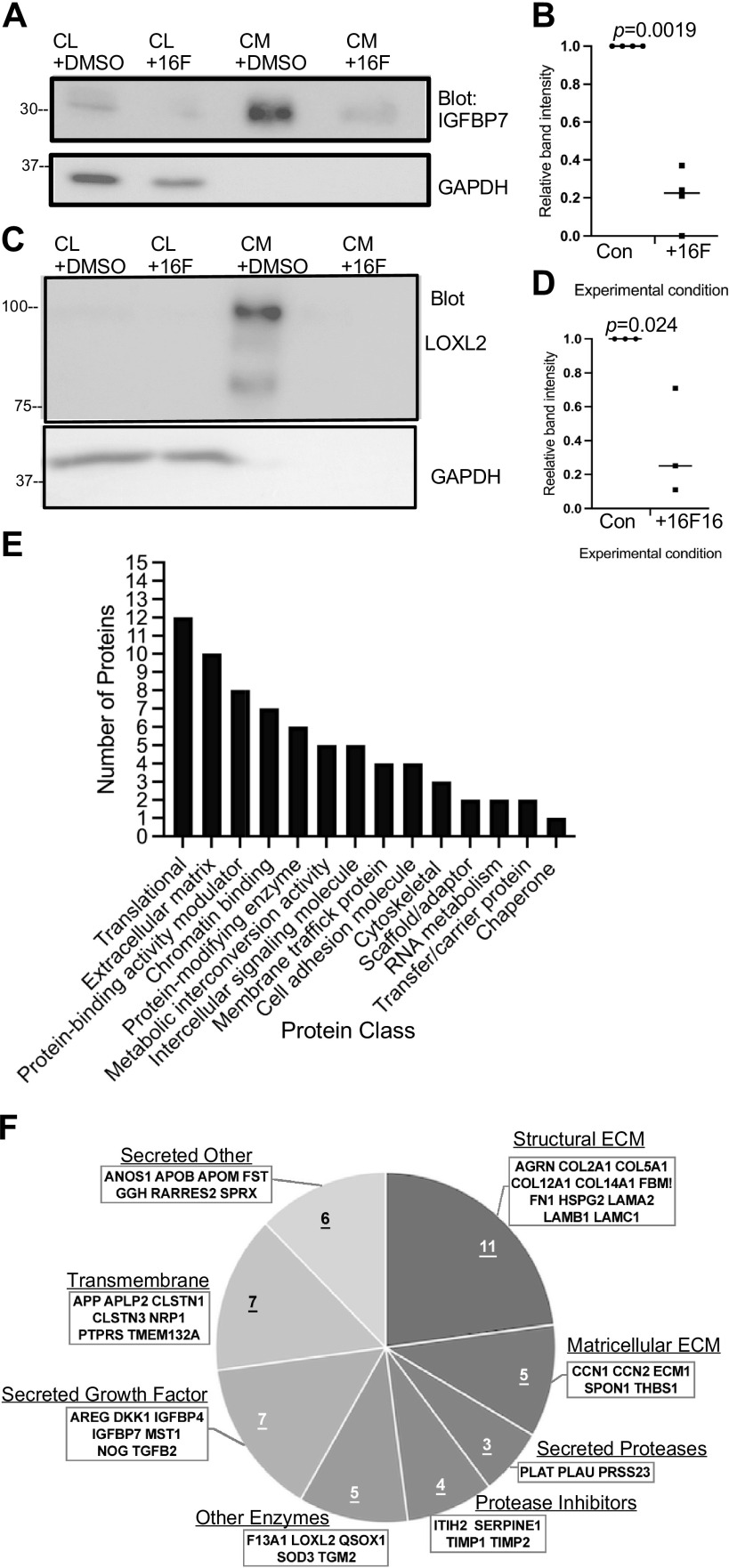
Analysis of the proteomics data set. *A* and *C*: validation by immunoblotting of effects of 16F16 treatment on IGFBP7 (*A*) or LOXL2 (*C*) abundance in cell lysates (CL) or conditioned media (CM) of MDA-MB-231 cells. GAPDH was used as a loading control. Molecular mass markers are given in kDa. *B* and *D*: quantified analysis of IGFBP7 or LOXL2 in CM from multiple independent experiments. For each blot and primary antibody, the normalized +DMSO band intensity was set as 1 and the band intensity of the +16F16 condition ratioed to the control (Con). Each data point represents 1 experiment; horizontal bars show the mean and SD. Statistical analysis by unpaired *t* test with Welch’s correction. *E*: analysis of protein classes in the 80-protein proteomics data set by PANTHER. *F*: molecular categorization of the extracellular proteins from the data set. Based on UniProt and NCBI GenPept. ECM, extracellular matrix.

Two proteins were chosen for experimental validation of the proteomics results by immunoblotting. Insulin-like growth factor binding protein 7 (IGFBP7) was chosen as a protein with a small fold change and significance close to the *P*-value cutoff (Fig. 3*B*, blue arrow). In addition, IGFBP7 is less abundant in the CM of *Pdia3*-null MEFs compared with CM from WT-MEFs (25). IGFBP7 was detected principally in CM, with minor amounts in cell lysate, as a band of the expected molecular mass of 30 kDa. IGFBP7 was confirmed to be more abundant in the CM of control, DMSO-treated cells (Fig. 4, *A* and *B*). The second protein examined was lysyl oxidase-like 2 (LOXL2). LOXL2 was among the proteins with the greatest fold decrease after 16F16 treatment, which scored with high significance (Fig. 3*B*, magenta arrow). LOXL2 has roles in collagen cross linking by oxidative deamidation of lysine residues (48) and has previously been identified as a PDIA3 substrate (49), and its abundance is quantitatively reduced in the CM of *Pdia3*-null MEFs compared with CM from WT-MEFs (25). All blots for LOXL2 (87 kDa) showed a band below 100 kDa and a second band ∼75 kDa in the CM, and very little LOXL2 was detected in cell lysates (Fig. 4*C*). As expected, LOXL2 was decreased in the CM of 16F16-treated cells (Fig. 4, *C* and *D*). Extracellular LOXL2 can be cleaved by serine proteases into an ∼65-kDa form (50), and the lower-molecular mass band likely corresponds to cleaved LOXL2.

### The Proteomics Data Set Is Highly Enriched in Proteins Relevant to the Extracellular Matrix

We categorized the 80 proteins of the proteomics data set for cellular location according to UniProt’s curated database and manual inspection. This identified 48 extracellular proteins, 30 intracellular proteins, and 2 proteins that are classified by UniProt as both intracellular and extracellular, TGLN2 and GGH. In further analyses, we included TGLN2 in the intracellular set and GGH in the extracellular set because of its NH_2_-terminal secretory signal peptide, as identified by SIGNALP 6.0. In validation of the experimental approach, the 80 proteins include known heparin-binding proteins among the extracellular proteins (Supplemental Table S2). The intracellular proteins are not known as heparin-binding proteins. The extracellular proteins also include known substrates of PDIA3 (Supplemental Table S2). Of the intracellular proteins, RNASE4 is known as a PDI substrate (51).

Seventy-one of the proteins from the proteomics data set could be grouped into fourteen PANTHER protein classes, with ribosomal proteins and extracellular matrix proteins being the most numerous classes (Fig. 4*E*). Categorization of the extracellular set of proteins by UniProt criteria confirmed that structural and matricellular ECM proteins are both present (Fig. 4*F*). The extracellular set also includes seven proteins with transmembrane domains: calsyntenins 1 and 3 (CLSTN1 and CLSTN3), which are members of the cadherin superfamily (52); neuropilin 1 (NRP1); protein tyrosine phosphatase receptor type S (PTPRS); TMEM132A; amyloid precursor protein (APP); and amyloid beta precursor like protein 2 (APLP2). The enzyme quiescin sulfhydryl oxidase 1 (QSOX1) also has a transmembrane domain, but QSOX1 is found mostly in the Golgi or is secreted in some contexts (53). The extracellular protein set also included proteases, protease inhibitors, secreted growth factors, and other enzymes and secreted proteins (Fig. 4*F*).

Next, we analyzed the proteomics data set of 80 proteins for enrichment in Gene Ontology (GO) gene sets. The “Biological Process” category identified enrichment for terms related to developmental processes, extracellular structures and the ECM, and also cytoplasmic translation. The “Molecular Function” category also emphasized enrichment for terms related to ECM, integrins and proteoglycans, but also signaling receptor binding and protein-containing complex binding. The “Cellular Component” category also identified significant enrichment for terms related to extracellular space and ECM, along with enrichment for exosome, extracellular vesicle, and extracellular organelle (Table 3). The analysis was repeated separately for the “Intracellular” and “Extracellular” protein groups. This analysis confirmed significant enrichment for intracellular proteins relevant to peptide and amide synthesis, ribosomal structure, and translation (Table 3). The intracellular proteins also showed enrichment for Molecular Functions of “cadherin binding” and “cell adhesion molecule binding” and for the Cellular Components of exosomes and extracellular vesicles. Enrichment of the extracellular proteins for Biological Process categories related to ECM organization and function was confirmed. The function of heparin binding was also enriched in this group. With regard to Cellular Components, the extracellular proteins were enriched for the terms “endomembrane system” and ‘ER lumen” as well as “vesicle” (Table 3). In general, GO enrichments for the extracellular set were of higher statistical significance than those associated with the full set of proteins or the intracellular set (Table 3; see FDR *q* values).

**Table 3.** Enriched Gene Ontology terms for the PDIA3-dependent, heparin-binding secretome of MDA-MB-231 cells

	No. in Set	No. in Overlap	No. Expected	Fold Enrichment	Raw *P* Value	FDR *q* Value
*All (80 proteins)*						
GO Biological Function						
Cell adhesion	964	22	3.75	5.87	1.30E−11	2.04E−07
Positive regulation of developmental process	1,314	24	5.11	4.70	1.16E−10	9.05E−07
Anatomical structure morphogenesis	2,185	30	8.49	3.53	2.70E−10	1.41E−06
Regulation of developmental process	2,463	31	9.57	3.24	1.01E−09	3.96E−06
External encapsulating structure organization	297	12	1.15	10.40	2.45E−09	4.80E−06
Extracellular structure organization	295	12	1.15	10.47	2.28E−09	5.10E−06
Tissue development	1,687	25	6.55	3.81	3.13E−09	5.45E−06
Cytoplasmic translation	124	9	0.48	18.68	2.27E−09	5.93E−06
Regulation of cell differentiation	1,568	24	6.09	3.94	3.83E−09	6.00E−06
Extracellular matrix organization	294	12	1.14	10.50	2.19E−09	6.88E−06
GO Molecular Function						
Structural molecule activity	729	29	2.83	10.24	1.22E−21	6.00E−18
Extracellular matrix structural constituent	173	18	0.67	26.78	2.31E−20	5.66E−17
Cell adhesion molecule binding	551	20	2.14	9.34	3.65E−14	5.97E−11
Glycosaminoglycan binding	241	15	0.94	16.02	5.32E−14	6.52E−11
Heparin binding	173	13	0.67	19.34	3.10E−13	2.53E−10
Sulfur compound binding	272	15	1.06	14.19	2.82E−13	2.77E−10
Proteoglycan binding	37	8	0.14	55.65	6.75E−12	4.73E−09
Signaling receptor binding	1,594	26	6.19	4.20	1.76E−10	1.08E−07
Integrin binding	159	10	0.62	16.19	1.03E−09	5.60E−07
Protein-containing complex binding	1,292	22	5.02	4.38	3.04E−09	1.49E−06
GO Cellular Component						
Extracellular space	3,419	67	13.28	5.04	2.95E−39	6.01E−36
Extracellular region	4,393	70	17.07	4.10	2.56E−36	2.61E−33
Collagen-containing extracellular matrix	430	29	1.67	17.36	8.53E−28	5.81E−25
External encapsulating structure	573	30	2.23	13.47	9.93E−26	4.05E−23
Extracellular matrix	572	30	2.22	13.50	9.46E−26	4.83E−23
Extracellular exosome	2,100	46	8.16	5.64	4.52E−25	1.54E−22
Extracellular membrane-bounded organelle	2,124	46	8.25	5.57	7.26E−25	1.65E−22
Extracellular organelle	2,124	46	8.25	5.57	7.26E−25	1.85E−22
Extracellular vesicle	2,123	46	8.25	5.58	7.12E−25	2.08E−22
Vesicle	3,964	54	15.40	3.51	9.74E−21	1.99E−18
*Intracellular (30 proteins)*						
GO Biological Function						
Peptide biosynthetic process	404	12	0.59	20.39	2.42E−13	1.26E−09
Cytoplasmic translation	124	9	0.18	49.81	1.87E−13	1.47E−09
Translation	377	12	0.55	21.85	1.09E−13	1.71E−09
Amide biosynthetic process	520	12	0.76	15.84	4.36E−12	1.71E−08
Peptide metabolic process	538	12	0.78	15.31	6.43E−12	2.02E−08
Cellular macromolecule biosynthetic process	764	12	1.11	10.78	3.44E−10	8.99E−07
Cellular amide metabolic process	803	12	1.17	10.26	6.02E−10	1.35E−06
Macromolecule biosynthetic process	1,291	14	1.88	7.44	8.61E−10	1.69E−06
Cellular nitrogen compound biosynthetic process	1,381	14	2.01	6.96	2.05E−09	3.58E−06
Nucleosome assembly	99	6	0.14	41.59	8.12E−09	1.27E−05
GO Molecular Function						
Nucleic acid binding	3,999	25	5.83	4.29	8.69E−14	4.26E−10
RNA binding	1,655	18	2.41	7.46	7.15E−13	1.75E−09
Structural constituent of ribosome	168	9	0.24	36.77	2.53E−12	4.13E−09
Cadherin binding	324	10	0.47	21.18	2.45E−11	3.01E−08
Heterocyclic compound binding	5,974	25	8.70	2.87	1.06E−09	1.04E−06
Organic cyclic compound binding	6,046	25	8.81	2.84	1.39E−09	1.14E−06
Cell adhesion molecule binding	551	10	0.80	12.46	3.78E−09	2.32E−06
Structural molecule activity	729	11	1.06	10.36	3.46E−09	2.43E−06
Protein heterodimerization activity	326	6	0.48	12.63	7.14E−06	3.89E−03
Ribonuclease A activity	4	2	0.01	>100	3.06E−05	1.50E−02
GO Cellular Component						
Cytosolic ribosome	104	9	0.15	59.39	4.19E−14	8.55E−11
Extracellular membrane-bounded organelle	2,124	20	3.09	6.46	2.16E−13	8.80E−11
Extracellular organelle	2,124	20	3.09	6.46	2.16E−13	1.10E−10
Extracellular vesicle	2,123	20	3.09	6.47	2.14E−13	1.45E−10
Extracellular exosome	2,100	20	3.06	6.54	1.74E−13	1.78E−10
Protein-containing complex	5,702	27	8.31	3.25	1.47E−12	5.00E−10
Cytosolic large ribosomal subunit	58	7	0.08	82.83	4.26E−12	1.24E−09
Ribosomal subunit	187		9	0.27	6.35E−12	1.62E−09
Extracellular space	3,419	22	4.98	4.42	1.09E−11	2.47E−09
Ribosome	226	9	0.33	27.33	3.23E−11	6.60E−09
*Extracellular (50 proteins)*						
GO Biological Function						
Anatomical structure morphogenesis	2,185	28	5.31	5.28	5.00E−15	3.92E−11
Cell adhesion	964	21	2.34	8.97	2.62E−15	4.11E−11
Positive regulation of developmental process	1,314	21	3.19	6.58	1.00E−12	3.93E−09
Regulation of developmental process	2,463	27	5.98	4.51	9.11E−13	4.76E−09
External encapsulating structure organization	297	12	0.72	16.64	7.49E−12	1.68E−08
Extracellular structure organization	295	12	0.72	16.75	6.94E−12	1.81E−08
Extracellular matrix organization	294	12	0.71	16.81	6.68E−12	2.09E−08
Tissue development	1,687	22	4.10	5.37	1.26E−11	2.48E−08
Multicellular organism development	4,129	32	10.03	3.19	1.88E−11	2.94E−08
Regulation of multicellular organismal development	1,356	20	3.29	6.07	1.80E−11	3.13E−08
GO Molecular Function						
Extracellular matrix structural constituent	173	18	0.42	42.84	1.53E−24	7.50E−21
Glycosaminoglycan binding	241	15	0.59	25.63	2.56E−17	6.29E−14
Sulfur compound binding	272	15	0.66	22.71	1.42E−16	2.32E−13
Heparin binding	173	13	0.42	30.94	4.49E−16	5.51E−13
Structural molecule activity	729	18	1.77	10.17	5.66E−14	5.55E−11
Proteoglycan binding	37	8	0.09	89.03	1.33E−13	1.09E−10
Signaling receptor binding	1,594	23	3.87	5.94	4.28E−13	3.00E−10
Integrin binding	159	10	0.39	25.90	8.03E−12	4.92E−09
Growth factor binding	134	7	0.33	21.51	4.67E−08	2.55E−05
Peptidase regulator activity	233	8	0.57	14.14	1.08E−07	5.31E−05
GO Cellular Component						
Collagen-containing extracellular matrix	430	29	1.04	27.77	2.11E−35	4.31E−32
External encapsulating structure	573	30	1.39	21.56	1.25E−33	8.48E−31
Extracellular matrix	572	30	1.39	21.60	1.19E−33	1.21E−30
Extracellular region	4,393	48	10.67	4.50	5.91E−30	3.02E−27
Extracellular space	3,419	45	8.30	5.42	9.07E−30	3.70E−27
Endoplasmic reticulum lumen	312	21	0.76	27.72	5.40E−25	1.84E−22
Endomembrane system	4,727	44	11.48	3.83	3.06E−22	8.91E−20
Basement membrane	98	11	0.24	46.22	1.67E−15	4.26E−13
Endoplasmic reticulum	2,024	27	4.92	5.49	7.95E−15	1.80E−12
Vesicle	3,964	34	9.63	3.53	9.34E−14	1.91E−11

Results are shown from PANTHER analysis of proteins in the proteomics data set that were downregulated by >2-fold at *q* = 0.05 for all 80 proteins (*top*), intracellular proteins only (*middle*), and extracellular proteins only (*bottom*). The top 10 Gene Ontology (GO) terms, ranked by false discovery rate (FDR) *q* value, are shown for each GO category.

We confirmed the GO enrichments assigned by PANTHER by analysis of the intracellular and extracellular sets of proteins with the separate tool, GOrilla, that identifies and statistically analyzes enrichment for GO terms in ranked lists of genes (34). The GOrilla analysis of the intracellular proteins indicated biological association with biosynthetic cellular processes, “RNA metabolic process,” and “negative regulation of gene expression” (Supplemental Fig. S1A) and also enrichment for the Molecular Function ‘nucleic acid binding” (Supplemental Fig. S1B). In the Cellular Components category, there was enrichment of proteins relevant to the large ribosomal subunit, protein-DNA complexes, and nucleosomes (Supplemental Fig. S1C). These results confirm an association of the identified intracellular proteins with protein translation and the ribosome, but by this method enrichment for cellular components of exosomes or extracellular vesicles was not specifically detected.

For the extracellular proteins, numerous GO terms were enriched with high statistical significance. In the category Biological Function, the most significant enrichments were for proteins involved in extracellular structure organization, biological and cell adhesion, multicellular organism development and organization, and posttranslational protein modification. Proteins associated with protein degradation, morphogenetic processes, and regulation of signaling receptor activity were also enriched, albeit with lower statistical significance (Supplemental Fig. S2A). Enriched Molecular Functions terms included ECM structural constituent, glycosaminoglycan binding, and heparin binding and broadly recapitulated the molecular functions identified by PANTHER (Supplemental Fig. S2B). Strong enrichment for Cellular Component terms of extracellular space or ECM and ER lumen also were confirmed (Supplemental Fig. 2C). Thus, the enriched GO terms identified by PANTHER and GOrilla were in broad agreement.

### Protein-Protein Interactions between Proteins of the Data Set

**Figure 5.**
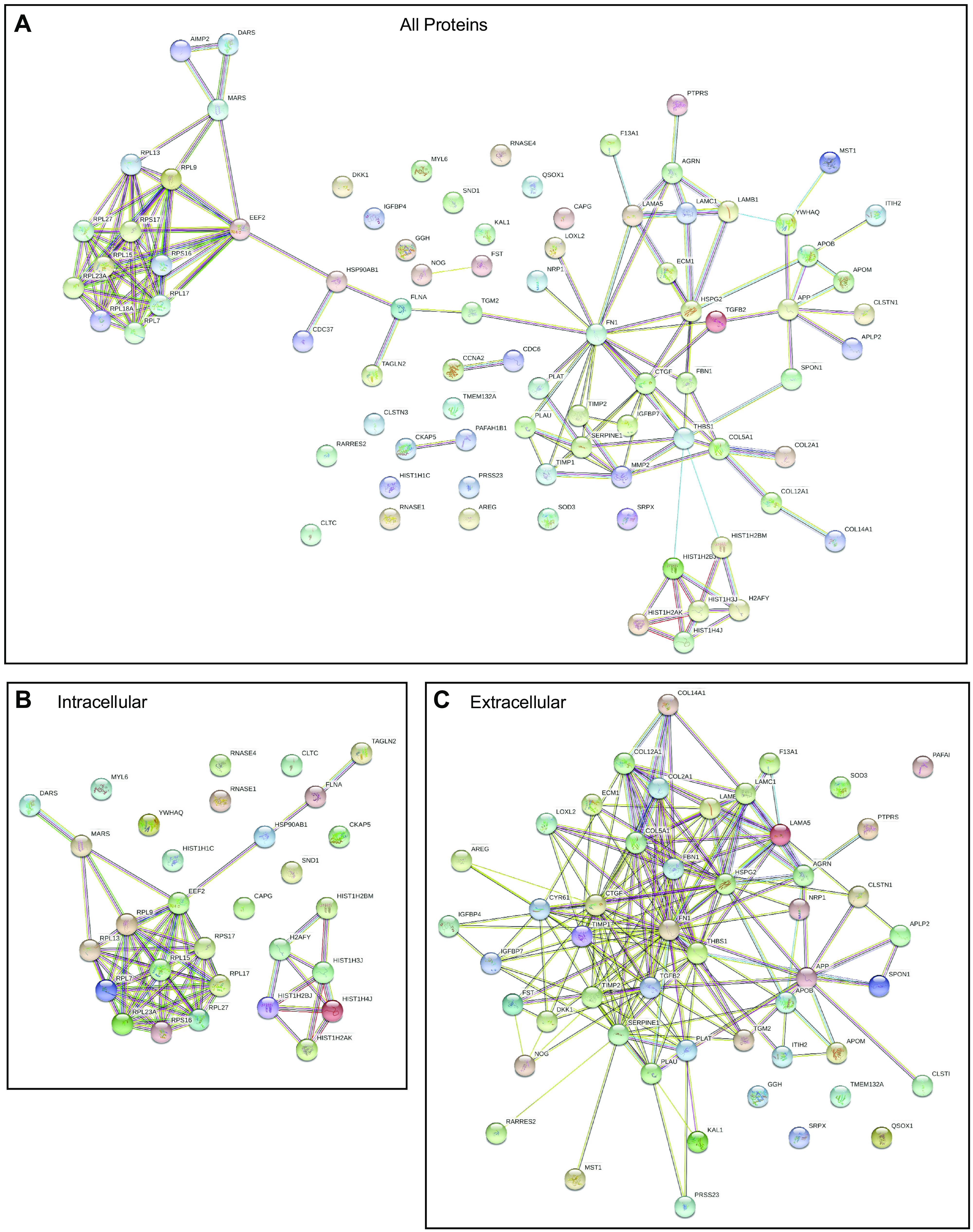
Known protein-protein interactions of proteins in the proteomics data set. *A*: STRING analysis for interactions within the entire proteomics data set (80 proteins). *B*: analysis of intracellular proteins only. *C*: analysis of extracellular proteins only. Each network contains only the query proteins. Evidence based on experiments, text mining, databases, and co-occurrence and high-confidence interaction score (0.7). Line thickness indicates the strength of supporting data.

Because the proteomics data set is enriched for protein classes and GO terms related to either ECM or ribosomal functions, it was relevant to examine the potential for protein-protein interactions between proteins of the data set. This question was addressed with the algorithm STRING (Search Tool for the Retrieval of Interacting Genes/Proteins) (38), which searches proteins of interest against a curated database of protein-protein interactions (PPIs) based on experimental and other criteria. STRING analysis of all 80 proteins produced a network with three distinct foci, corresponding to a ribosomal protein cluster, a histone protein cluster, and an extensive network that included ECM proteins, proteases, protease inhibitors, and signaling proteins. This network was linked to the ribosomal cluster through interactions of proteins such as HSP90AB1. Twenty-one proteins had no known interactions with the others (Fig. 5*A*). A separate analysis of the intracellular proteins recapitulated the two clusters of ribosomal proteins and histone proteins; these clusters were not linked together (Fig. 5*B*).

Analysis of only the extracellular proteins showed that many of them participate in a network of interactions, with FN1, HSPG2, and APP forming prominent nodes in the network (Fig. 5*C*). A subnetwork including plasminogen activator, tissue type (PLAT), plasminogen activator, urokinase (PLAU), Serpin family E member 1 (SERPINE1), and tissue inhibitors of metalloproteinases (TIMPs) relates to proteolytic regulation and accounted for the enrichment in GO terms “peptidase regulator activity” and “platelet alpha granule lumen” (Table 3 and Supplemental Fig. S2). A subnetwork centered on HSPG2 and including laminin subunit alpha-5 (LAMA5), laminin subunit beta-1(LAMB1), and laminin subunit gamma-1(LAMAC1) accounted for the enrichment of GO terms related to the basement membrane. Many other interacting proteins such as CCN2 (CTGF) and TGFB2 also relate to ECM organization and turnover (Fig. 5*C*).

### Disulfide-Bonded Domains and β-Hairpin Folds Are Prominent in Extracellular Proteins of the Data Set

PDIA3 is an oxidoreductase and disulfide isomerase that acts in protein folding and refolding (49). We examined the domain composition of the 80 proteins of the proteomics data set to identify the proteins with at least one disulfide bond or disulfide-bonded domain. Nearly all the extracellular proteins in the data set contain disulfide bonds, with the exception of SERPINE1 (Supplemental Table S2). Although disulfides in Transmembrane Protein 123 (TMEM123A) were not identified by these criteria, conserved cysteines identified within its extracellular immunoglobulin-like domains are suggested to form intramolecular disulfides (54). The most common disulfide-containing domain was the epidermal growth factor (EGF) domain. Of the intracellular proteins in the data set, Ribonuclease A family member 4 (RNASE4) contained disulfide bonds and is a known PDI substrate (51). We therefore focused on the extracellular proteins for further analysis of the disulfide-bonded domains.

Information on the structural fold families to which the disulfide-bonded domains belong was collected from the Structural Classification of Proteins (SCOP) database and the curated Swiss-Prot section of UniProt. In total, domains belonging to 45 fold families that each contain at least one conserved disulfide bond were identified. Of these, seven disulfide-containing folds were of particular interest because they are represented in many proteins of the data set (Table 4). Furthermore, these folds were also represented in the PDIA3-dependent proteins identified in our previous proteomic comparison of the heparin-binding secretomes of WT-MEFs and *Pdia3*-null MEFs (25) (Table 4). These folds contained between one and three conserved disulfides, and the most common fold was the knottin fold: indeed, the EGF domain family is included in the knottin fold superfamily.

**Table 4.** Identification of the most common disulfide-bonded fold types in proteins of the proteomics data set from MDA-MB-231 cells

Fold/Superfamily (SCOP ID No.)	Total No. of Unique Proteins (from both MEF and MDA-MB-231 samples)	Number of β-Strands in Fold	Number of Conserved Disulfide Bonds in Fold	Proteins from MEF Proteome*	Proteins from MDA-MB-231 Proteome
Knottin (2000181)	17	3	3	**CCN2, IGFBP7**, NRG1, NRG2	AGRN, ANOS1, AREG, FBN1, FST, HSPG2, CCN1, **CCN2**, IGFBP4, **IGFBP7**, LAMA5, LAMB1, LAMC1, PLAT, THBS1
Ig-like Beta Sandwich (2000051)	14	7–9	1	CDON, **IGFBP7**, NRG2	ANOS1, CLSTN1, CLSTN3, COL12A1, COL14A1, F13A1, FN1, HSPG2, **IGFBP7**, PTPRS, SRPX, TGM2
FN1-like (3000979)	7	5	2	BMPER, **CCN2**, COL1A1	CCN1, **CCN2**, COL2A1, FN1, THBS1
Cystine Knot Cytokines (2000537)	5	4	3	INHBA, INHBB, VEGFA	NOG, TGFB2
Kazal (2000404)	3	3	3	**IGFBP7**	AGRN, FST, **IGFBP7**
Kunitz inhibitors (4002025)	2	2	3	**APP**	APLP2, **APP**
Scavenger receptor cysteine-rich (SRCR)-like (2000834)	1	7	3–4	**LOXL2**	**LOXL2**

Domains identified by InterProScan or CDD were assigned to fold families in SCOP. *The same searches were carried on heparin-binding proteins of altered abundance in the conditioned medium (CM) of *Pdai3*^−/−^ mouse embryo fibroblasts (MEFs) compared with the CM of wild-type MEFs, as identified by tandem mass tagging (TMT) proteomics in Ref. 25. Proteins identified from both proteomes are in bold.

**Figure 6.**
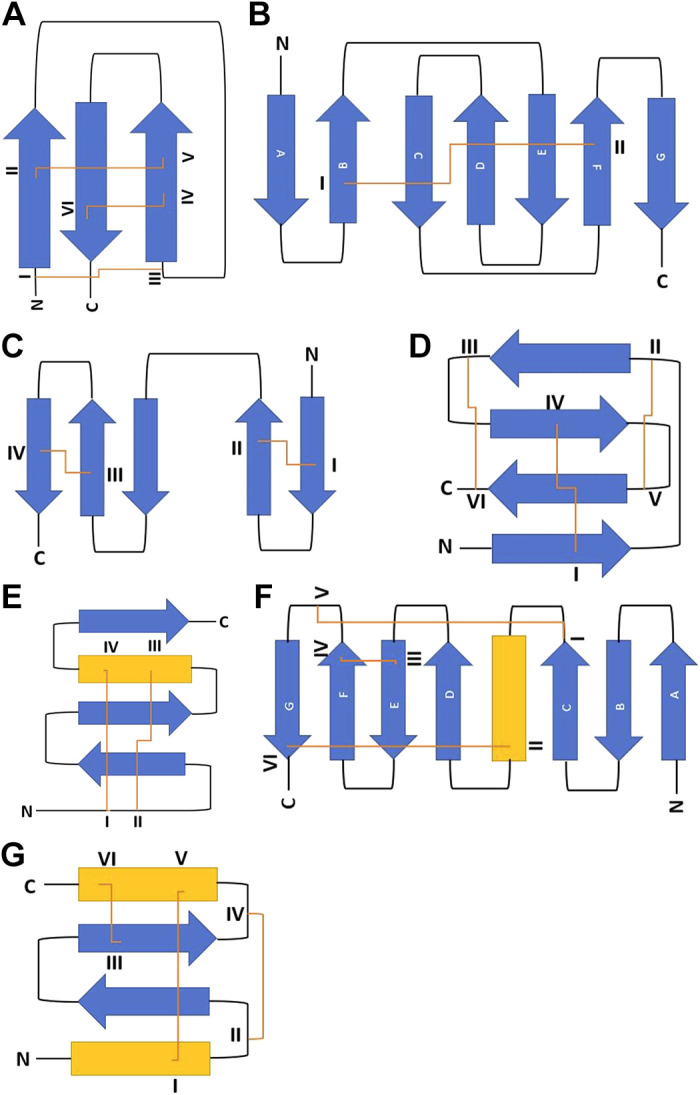
Common structural folds present in extracellular proteins of the proteomics data set. Protein domains were identified in InterProScan and/or CDD and GenPept and fold categories assigned in SCOP. *A*: knottin fold. *B*: Immunoglobulin-like beta-sandwich fold. *C*: Fibronectin type I domain. *D*: Cysteine knot cytokine fold. *E*: Kazal fold. *F*: Scavenger receptor cysteine-rich (SRCR)-like fold. *G*: Kunitz inhibitor fold. In each panel, cysteine residues are numbered in roman numerals in sequence from the NH_2_ terminus (N) to the COOH terminus (C). Disulfide bonds are shown as orange lines. β-Strands are in blue, and α-helices are in yellow.

All seven of these disulfide-containing folds consist mostly, if not entirely, of antiparallel β-strands forming two or more β-hairpins (Fig. 6). The β-hairpin structure consists of two antiparallel β-strands linked by a turn (55). Within the general category of β-hairpins, the knottin fold and cysteine knot cytokine (CKC) fold form cysteine knot motifs (CKMs), in which multiple disulfide bonds are not sequential but intertwined (56) through different cysteine linkage patterns (Fig. 6, *A* and *D*). Overall, the prevalence of β-hairpin folds in the common disulfide-bonded domains suggests that β-hairpin structures and in particular the knottin fold may be relevant to the substrate specificity of PDIA3.

### Expression of the Extracellular Protein Set Correlates with the Basal Subtype of Breast Cancer and Decreased Metastasis-Free Survival

**Figure 7.**
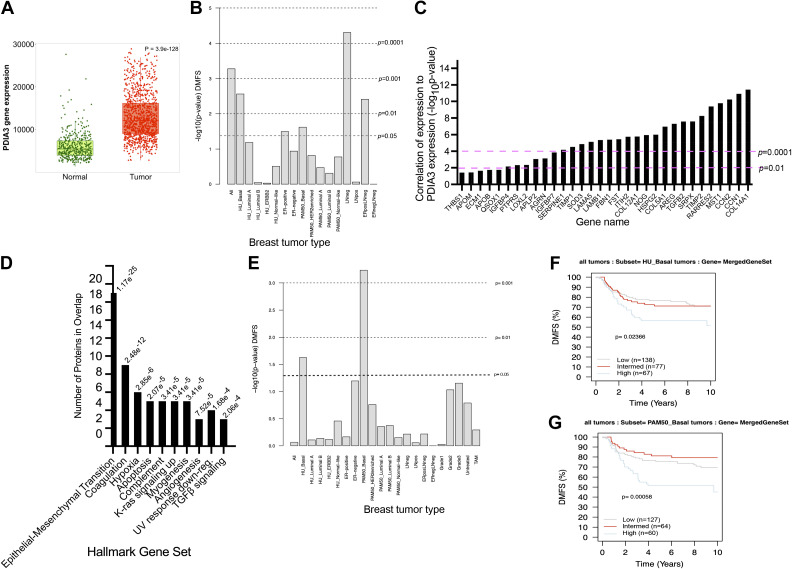
Relevance of PDIA3 and the extracellular proteins from the proteomics data set to breast cancer and survival outcomes. *A*: elevated expression of *PDIA3* in invasive breast cancers vs. normal tissue. Data from RNASeq data sets in the TNMplot database. Horizontal bars in the boxplots represent the median. Statistical analysis by Mann–Whitney *U* test. *B*: correlation of *PDIA3* with reduced distant metastasis-free survival (DMFS) in human breast cancers. Each column represents a different data set in the GOBO breast cancer database; dashed lines show the indicated *P* values. *C*: transcripts encoding extracellular proteins from our proteomics data set that correlate (at *P* value < 0.05) with expression of PDIA3 in invasive breast tumors. From RNAseq data in the TNMplot database. *D*: enrichment overlap of the extracellular proteins with Hallmark gene sets in the GSEA Molecular Signatures database. The FDR *q* value is shown for each overlap gene set. *E*: correlation of the extracellular proteins with reduced DMFS in basal-type breast cancers. Each column represents a different data set in the GOBO breast cancer database; dashed lines show the indicated *P* values. *F* and *G*: Kaplan–Meier plots of patient DMFS over a 10-yr period for patients with low (gray), intermediate (red), or high (blue) expression of the extracellular proteins, analyzed in GOBO as a merged gene set. *F*: plot based on basal tumor data set from Ref. 58. *G*: plot based on basal tumor data set from Ref. 57.

Several publications have implicated PDIA3 with aggressive breast cancer (see introduction). In an analysis of *PDIA3* gene expression based on RNASeq data in the TNMplot database, we confirmed that PDIA3 expression is typically elevated in invasive breast cancers versus normal tissue (Fig. 7*A*). With regard to clinical outcomes, *PDIA3* expression was searched against the breast cancer data sets in the GOBO database (42) and found to correlate with significantly reduced (*P* value <0.05) distant metastasis-free survival (DMFS) over 10 yr for all tumors; basal subtype tumors; estrogen receptor (ER)-positive tumors; lymph node-negative tumors, and ER-positive, lymph node-negative tumors (Fig. 7*B*). We also examined whether expression of *PDIA3* correlated with expression of any of the transcripts encoding the extracellular proteins from our proteomics data set, through the “correlation analysis” tool of TNMplot. Expression of 32 of the transcripts correlated significantly (*P* value <0.05) with *PDIA3* expression (Fig. 7*C*). Eighteen of these encoded proteins contain the most common disulfide-bonded folds (see Table 4), and most of the encoded proteins, with the exception of QSOX, SOD3, and SRPX, are participants in extracellular protein-protein interaction networks (see Fig. 5).

Experimentally, we have shown that the PDIA3 inhibition inhibits MDA-MB-231 cell spreading (present study) and migration (23). The identified GO enrichments and PPI networks for the extracellular proteins in our data set emphasize their roles in processes relevant to breast cancer metastasis, such as ECM organization, cell attachment, and cell movement. We therefore examined whether the extracellular proteins in our proteomics data set correlate with known cancer-associated gene signatures. From the Gene Signature Expression Analysis (GSEA) database of “Hallmark gene sets,” the most significant match was with the gene set “Epithelial-mesenchymal transition.” Other gene set matches of obvious relevance to cell biological processes of cancer included “Hypoxia,” “Apoptosis,” “K-ras signaling up-regulated,” and “Angiogenesis” (Fig. 7*D*). When the 15 knottin fold-containing proteins (see Table 4) were used as the query, the top-scoring match was also with the gene set “Epithelial-mesenchymal transition” (7 proteins in the gene set overlap, with FDR *q* value of 1.91E−11).

It was thus of interest to investigate whether the extracellular proteins themselves correlate with specific molecular subtypes of breast cancer or patient outcomes. The complete list of 80 proteins was searched as a merged gene set against the GOBO database with regard to breast cancer molecular subtypes and patient outcomes for DMFS over 10 yr. The complete set of proteins correlated weakly with reduced DMFS for patients with ER-negative tumors (*P* = 0.051) and with basal-type tumors [as categorized by the PAM50 data set (57), *P* = 0.02] (not shown). Analysis with only the extracellular proteins as a merged gene set yielded significant correlation of expression with reduced DMFS for the basal breast cancer subtype in both the PAM50 (57) and Hu (58) data sets (Fig. 7*E*). Kaplan–Meier plots confirmed that highest expression levels of the merged gene set correlated with reduced DMFS of patients in both basal subtype data sets (Fig. 7, *F* and *G*).

## DISCUSSION

To our knowledge, this is the first examination by proteomics of the effects of 16F16 inhibition on secreted proteins of MDA-MB-231 breast cancer cells. Starting from heparin-binding proteins in CM, because of the role of these proteins in supporting effective spreading and F-actin organization by MDA-MB-231 cells, we have identified 80 proteins that are consistently decreased in abundance in CM after 16F16 treatment. Our analyses of these proteins demonstrate complexity in the data set, which includes both extracellular and intracellular proteins. We also establish important commonalities of functional enrichments, protein-protein interactions, and, for the extracellular proteins, commonalities of domain structure. Focusing in on the extracellular proteins, we interrogated breast tumor gene expression data sets to show that high expression of this gene set correlates with reduced distant metastasis-free survival of patients with basal subtype breast cancer. Thus, PDIA3 inhibition may offer a relevant new strategy to apply to this hard-to-treat subtype of breast cancer.

Our proteomic data set included extracellular glycoproteins previously identified as substrates of PDIA3 such as laminin beta1 or LOXL2 (49, 59) and additional proteins not previously connected with PDIA3 function. The prior studies identified the PDIA3 substrates to include certain glycoproteins of the ECM, and indeed GO analysis of our data set showed clear enrichment of proteins related to ECM structure, function, or extracellular proteolytic activities. Another substrate-trapping study identified proteins in the lectin pathway of complement activation as PDIA3 substrates (60), and our proteomics set also included proteins relevant to the complement cascade, such as TIMP, PLAU, and PLAT. Although 16F16 inhibits, to a lesser extent, PDIA1 as well as PDIA3 (24), the relevance of our results to PDIA3 inhibition is shown by the inclusion of known PDIA3 substrates as well as by reidentification of several proteins (e.g., CCN2, IGFBP7) identified as PDIA3 dependent in our prior proteomics comparison of CM from WT-MEFs versus CM from *Pdia3*^−/−^ MEFs (25). As expected from our methodology, the majority of the extracellular proteins are known heparin-binding proteins (Supplemental Table S2) and ‘heparin binding” was among the enriched GO terms for molecular function.

Intracellular proteins also made up a substantial fraction of our identified set of proteins. In addition to proteins associated to ribosomes, the enriched GO terms for the intracellular proteins included “cadherin binding” and “cell adhesion molecule binding.” It is plausible that the correlated proteins (which include filamin A, CKAP5, and transgelin-2 as well as several ribosomal proteins) may have copurified in association with other, heparin-binding proteins; for example, filamin A has capacity for interaction with ribosome-associated proteins and with transglutaminase 2 (Fig. 5*A*). Furthermore, “exosomes” and “extracellular vesicles” were in the top 10 significantly enriched GO Cellular Components terms for both the complete proteomics data set and the intracellular protein subset, as identified by PANTHER. Indeed, many of the ribosomal or DNA-binding proteins in the data set are known constituents of extracellular vesicles such as exosomes, and MDA-MB-231 cells are known to produce exosomes (61, 62). Since our experimental method involved a 0.22-µm pore size filtration cutoff, it is likely that ∼100-nm-diameter extracellular vesicles would remain within the CM and could be retained on heparin-agarose through the surface exposure of heparin-binding proteins. This interpretation points to depletion of exosomal contents as another consequence of PDIA3 inhibition, in addition to effects on directly secreted proteins.

Because of the activities of PDIA3 as a catalyst of disulfide bond oxidation, reduction, and isomerization, we examined the proteins in the proteomics data set for the presence of intramolecular disulfide bonds according to biochemical and structural data. Strikingly, disulfides were concentrated in the extracellular proteins within the data set. Only one intracellular protein, the known PDI substrate RNASE4, contained disulfide bonds. PDIA3 specifically targets proteins with mono-glucosylated, N-linked oligosaccharide side chains through their association with the ER-resident chaperone proteins calreticulin and calnexin. By binding the P domains of calreticulin and calnexin, PDIA3 is brought into proximity with the substrate (63, 64). However, calreticulin and calnexin may also make protein-protein contacts with substrates in addition to the essential protein-carbohydrate cargo recognition mechanism (65).

Jessop et al. (49) noted that their set of identified PDIA3 substrates were enriched for small, disulfide-rich domains many of which were low in secondary structure. We identified here that the EGF domain [a small (∼50 residues) disulfide-rich domain] and more generally the knottin fold (the fold family that includes EGF domains) is the most frequent domain within the extracellular proteins of our proteomics data set. Functionally important N-glycosylation sites are known in some EGF domains (e.g., Ref. 66), and EGF domains also undergo unusual, O-linked carbohydrate modifications (67). The knottin fold comprises a disulfide-bonded β-hairpin structure that has various faces for protein-protein interactions and is very stable once fully folded (68). The identified commonality of knottin fold structure raises a novel implication for future study, that cysteine residues in the context of this fold may be preferred substrates for PDIA3 catalytic activity.

Laboratory data have shown a functional role of PDIA3 in breast cancer in vivo in mice (18, 22) or in promoting human breast cancer cell adhesion, spreading, and migration in vitro (Ref. 23 and present study). This proteomics study provides new insights into the molecular basis for these functional effects of PDIA3. The proteomics data set is strongly enriched for proteins related to ECM organization and proteolysis, and the ECM is an important component of the tumor microenvironment. The transmembrane proteins identified have roles in cell interactions and signaling. Several of these proteins (e.g., APP) are acted on by sheddases that release the extracellular domains, or portions thereof, to the extracellular milieu (69), and further studies will be needed to determine whether it is these fragments that are specifically downregulated by PDIA3 inhibition. It will also be of interest to determine whether these proteins are direct substrates of PDIA3. Furthermore, as identified by protein-protein interaction analysis, many of the proteins in our data set are known interaction partners within the ECM or at cell surfaces. The extracellular proteins from the proteomics data set also overlapped significantly with gene signatures characteristic of epithelial-mesenchymal transition. Thus, PDIA3 activity appears important for assembly of protein-protein networks that function to structure the ECM and in communications between normal and tumor cells that support tumor progression. This demonstrates the relevance of PDIA3 as a prospective new molecular target in breast cancer.

It is of interest that many of the extracellular proteins in the proteomics data set are also substrates of FAM20C kinase. FAM20C is a serine kinase that phosphorylates protein substrates in the secretory pathway (70) and is the major kinase for phosphorylation of extracellular phosphoproteins. Analysis of FAM20C substrates in CM of several cell lines has identified a wide range of proteins (71), which include IGFBPs, collagen, laminin subunits, CCN1, FN1, TIMP, ITIH2, PRSS23, APP, TMEM132A and QSOX1, which also feature in our data set. FAM20C has a functional association with PDIA3, through its phosphorylation and activation of endoplasmic reticulum oxidoreductase 1a (ERO1a) in the trans-Golgi network. Activated ERO1a binds to a folding chaperone, ERp44, that transports active ERO1a to the ER, where it interacts with PDIA3 to regulate its disulfide isomerase activity (48, 77). After interacting with PDIA3, inactivated ERO1 returns to the Golgi network. This PDI/ERO cycle has a crucial role in efficiently removing nonnative disulfide bonds formed during initial protein folding. Given the overlap between the extracellular proteins in our proteomics data set with known FAM20C substrates, it is of interest to note that a new small-molecule inhibitor of FAM20C proved effective to inhibit MDA-MB-231 migration in vitro and tumor growth in mice (72).

Based on breast cancer data sets in the TMNplot and GOBO databases, we confirmed that *PDIA3* expression is elevated in invasive breast tumors and correlated with the clinical outcome of DMFS time for several breast cancer subtypes, confirming its relevance as potential new target. Since investigations to date have concentrated on whole tumor analyses by proteomics or transcriptomics, the cell types that express *PDIA3* remain uncertain. From our observational immunohistochemical study on primary breast tumors (23), PDIA3 staining was apparent in the epithelial glandular structures, but not in the stroma, of a luminal-type tumor and generalized PDIA3 staining was detected in a basal-type tumor. Thus, tumor cells themselves express *PDIA3*, but further analyses with cell type-specific markers will be needed to determine its expression in tumor-associated cell types. From analysis of the set of extracellular proteins in our data set against breast cancer gene expression data sets, we identified a specific correlation between high expression of these gene products and reduced DMFS of patients with basal subtype breast cancer within a 10-yr period. Indeed, MDA-MB-A231 cells were derived from the pleural effusion of a basal tumor, have undergone EMT, and by transcriptomics have similarities to the basal-like molecular subtype of breast cancers (43, 73). The correlation of *PDIA3* with DMFS in several breast cancer subtypes, whereas transcripts encoding the extracellular proteins from our data set correlated specifically with poor DMFS outcomes in basal-type breast cancer, may suggest that PDIA3 has a different substrate repertoire in other molecular subtypes. Overall, the correlations of PDIA3 and transcripts encoding the extracellular proteins from our data set with DMFS in basal-type breast cancer implicate a clinical relevance to utilizing PDIA3 inhibition to reduce levels of these proteins. This would be predicted to counter EMT and reduce tumor cell motility/invasion. Moreover, basal subtype cancers are most commonly triple negative, i.e., negative for estrogen and progesterone receptors and human epidermal growth factor receptor 2 (HER2) (74). Basal-type tumors are not susceptible to adjuvant therapies such as tamoxifen (that block estrogen receptor function) or HER2 blockade and thus have limited treatment options and often have a poor prognosis (75). In summary, this study provides new insight into extracellular proteins of breast cancer cells that depend on PDIA3 activity for their production and shows a clinical relevance of these proteins to metastasis-free survival for patients with basal-type breast cancer, which is frequently an intractable form of breast cancer.

## SUPPLEMENTAL DATA

10.6084/m9.figshare.21222152 Supplemental Tables S1 and S2 and Supplemental Figures S1 and S2: https://doi.org/10.6084/m9.figshare.21222152.

## GRANTS

This research was supported by School of Biochemistry project support for A.G. and R.A. The Orbitrap Fusion Tribrid mass spectrometer was part-funded (50%) by a Wellcome Trust Multi-User Equipment grant (grant-holder Prof. Pete Cullen, University of Bristol, grant no. 104914/Z/14/Z).

## DISCLOSURES

No conflicts of interest, financial or otherwise, are declared by the authors.

## AUTHOR CONTRIBUTIONS

A.G., R.A., and J.C.A. conceived and designed research; A.G., K.J.H., and R.A. performed experiments; A.G., K.J.H., R.A., and J.C.A. analyzed data; A.G., K.J.H., R.A., and J.C.A. interpreted results of experiments; A.G., R.A., and J.C.A. prepared figures; J.C.A. drafted manuscript; A.G., K.J.H., and J.C.A. edited and revised manuscript; A.G., K.J.H., R.A., and J.C.A. approved final version of manuscript.
